# Nodular Regenerative Hyperplasia: A Case of Rare Prognosis

**DOI:** 10.1177/2324709617690742

**Published:** 2017-03-28

**Authors:** Mindy Lee, Manhal Izzy, Ashwin Akki, Kathryn Tanaka, Harmit Kalia

**Affiliations:** 1 Division of Gastroenterology and Liver diseases, Montefiore Medical Center, The Albert Einstein College of Medicine Bronx, NY, USA; 2 Division of Surgical Pathology, Montefiore Medical Center, The Albert Einstein College of Medicine Bronx, NY, USA

**Keywords:** Nodular regenerative hyperplasia, Hepatoma, Hepatocellular carcinoma, Noncirrhotic portal hypertension

## Abstract

**Introduction:** Nodular regenerative hyperplasia (NRH) is a known etiology of noncirrhotic portal hypertension. Cases of biopsy-proven NRH in human immunodeficiency virus (HIV)–positive patients have been described. While these patients often have normal synthetic liver function, several reports described disease progression to liver failure. **Case:** We here present a 26-year-old woman with history of congenital HIV on antiretroviral therapy complicated by *Pneumocystis carinii* pneumonia at age 14. CD4 counts have been >300 with undetectable viral load. She was referred to our Hepatology service for evaluation of splenomegaly, elevated liver tests, and thrombocytopenia. On initial presentation, she reported easy bruising and gingival bleeding, and abdominal imaging showed evidence of portal hypertension without associated cirrhosis. Upper endoscopy was significant for large esophageal varices without bleeding stigmata. Liver biopsy showed minimal fibrosis around the portal areas without significant inflammation. The lobules showed focal zones of thin hepatocyte plates on reticulin stain with adjacent areas showing mild regenerative changes. The diagnosis of NRH was made and patient was placed on propranolol for variceal bleeding prophylaxis. Two years later, the patient presented with bleeding gastric varices warranting transjugular intrahepatic portosystemic shunt. Postprocedure course was complicated by mild encephalopathy. Subsequent magnetic resonance imaging showed a 1.7 × 1.3 cm lesion suggestive of hepatocellular carcinoma (HCC). The patient was deemed to be a candidate for liver transplantation, and she is now delisted due to ongoing pregnancy. **Conclusion:** This report describes the first case of HCC in an HIV patient with NRH. The possible association of NRH with HCC warrants further investigation.

## Introduction

Nodular regenerative hyperplasia (NRH) is an increasingly recognized form of noncirrhotic portal hypertension (NCPH). It is a form of liver disease characterized by mostly preserved liver function, with signs of portal hypertension through clinical, radiologic, or endoscopic evaluation.^1^ Microscopically, NRH is characterized by fine nodules 1 to 3 mm in diameter distributed evenly throughout the liver. There are 2 populations of hepatocytes noted on the liver histology of these patients: centrally located hypertrophic hepatocytes and peripherally located atrophic hepatocytes. There is usually minimal to no fibrosis in the perisinusoidal or periportal areas. No fibrous septa are seen between the nodules, in contrast to the typical cirrhotic liver. These finding are best seen with reticulin staining.^2^

NCPH accounts for about 10% of cases of portal hypertension, and NRH accounts for 14% to 27% of all NCPH cases.^2^ Up to 50% of patients with NRH have evidence of portal hypertension, and the prognosis in these cases is largely dependent on presence and severity of portal hypertension.^3^ The pathogenesis of NRH is thought to be a compensatory hypertrophic response to neighboring areas of relative ischemia, with a component of hypercoagulability causing microthrombosis of the portal vasculature.^1^ NRH is postulated to be the most common cause of portal hypertension associated with hematologic disorders.^4^ It has been associated with different myeloproliferative disorders, for example, polycythemia vera and chronic myelogenous leukemia, as well as lymphoproliferative disorders, for example, Hodgkin’s lymphoma, non-Hodgkin’s lymphoma, and leukemia.^4^ Furthermore, it is associated with autoimmune and rheumatologic disorders. Certain medications have been associated with NRH; growing evidence has shown the relationship between NRH and use of azathioprine in the treatment of inflammatory bowel disease, thioguanine/busulfan in the treatment of leukemia,^5,6^ and antiretroviral medications in patients with human immunodeficiency virus (HIV; Table 1).^1^

**Table 1.** Antiretroviral Medications Associated With Nodular Regenerative Hyperplasia.

Medication Name	Class
Didanosine	Nucleoside reverse transcriptase inhibitor
Zidovudine	Nucleoside reverse transcriptase inhibitor
Stavudine	Nucleoside reverse transcriptase inhibitor
Tenofovir	Nucleotide reverse transcriptase inhibitor

Liver disease is known to cause significant morbidity and mortality in HIV seropositive patients.^1^ Coinfection with viral hepatitis remains the leading cause of liver disease in HIV patients. NRH is a less common cause of liver disease in these patients. It has been established that certain antiretroviral medications can lead to the development of NRH.^1,3,5^ The pathogenesis of NRH in HIV seropositive patients is not entirely clear. It has been hypothesized to be most likely drug-induced, and less convincingly viral or immune-mediated.

NRH can present with a wide spectrum of clinical symptoms; however, high clinical suspicion is the key to early diagnosis as most patients are asymptomatic.^1^ While some patients present with nonspecific symptoms such as chronic fatigue and weight loss, variceal bleeding can be the first presenting symptom in others. Wanless et al addressed the largely asymptomatic nature of this disease. His group found that out of 2500 autopsies, 64 patients had evidence of NRH, and of those, only 1 patient had documented esophageal varices ante mortem.^7^

With regard to NRH among HIV patients, to date, only about 95 cases of NRH have been reported among which the most common manifestations of portal hypertension were ascites, esophageal varices, hepatic encephalopathy, splenomegaly, and portal vein thrombosis. There has been no documented de novo hepatocellular carcinoma (HCC) in this population.^1^

HCC is the third most common cause of cancer-related deaths worldwide. It usually develops in patients with cirrhosis secondary to viral, autoimmune, or metabolic disorders. However, 15% to 20% of all cases of HCC can arise without cirrhosis or with minimal portal fibrosis.^8^ These patients were identified to be infected with hepatitis B virus (HBV), suggesting an oncogenic relationship between HCC and HBV.^9^ Limited data are available about the development of HCC in patients with NRH. Liver transplantation remains a therapeutic option with the excellent survival in patients with HCC and portal hypertension.^10,11^

We present a case of a patient with retroviral infection and NRH who had an unusual presentation and a rare prognosis.

## Case Description

A 26-year-old woman had a history of vertically transmitted HIV mono-infection on antiretroviral therapy complicated by oral thrush and *Pneumocystis carinii* pneumonia at age 14. She had been maintained on emtricitabine-tenofovir, atazanavir, and ritonavir. Her other medical problems included iron deficiency anemia and mild intermittent asthma. In addition to antiretroviral therapy, her other medications included albuterol and iron supplementation. Family history was significant for HIV infection in patient’s mother and oropharyngeal cancer in her aunt. No family history of liver disease or liver malignancy was reported. Social history did not reveal substance abuse. She was referred to hepatology for evaluation of splenomegaly, elevated liver tests, and thrombocytopenia. On initial evaluation, she reported easy bruising and gingival oozing. Physical exam revealed firm and enlarged spleen 10 cm below the costal margin with mild tenderness and without associated hepatomegaly. Initial laboratory results showed albumin of 3.9 g/dL, total protein of 7.7 g/dL, total bilirubin of 0.7 mg/dL, direct bilirubin of 0.2 mg/dL, blood urea nitrogen level of 11 mg/dL, creatinine level of 0.6 mg/dL, and glomerular filtration rate of >60 mL/min. Alkaline phosphatase was 260 U/L, aspartate aminotransferase 80 U/L, and alanine aminotransferase 83 U/L. Platelet count was 72 000/µL. International normalized ratio was 1.2. Other laboratory results showed iron deficiency anemia with hemoglobin of 9.6 g/µL. CD4 count was >300, and viral load was undetectable. Hepatitis B and C serology testing was unrevealing. Antinuclear and anti–smooth muscle antibodies were not detected.

**Figure 1.**
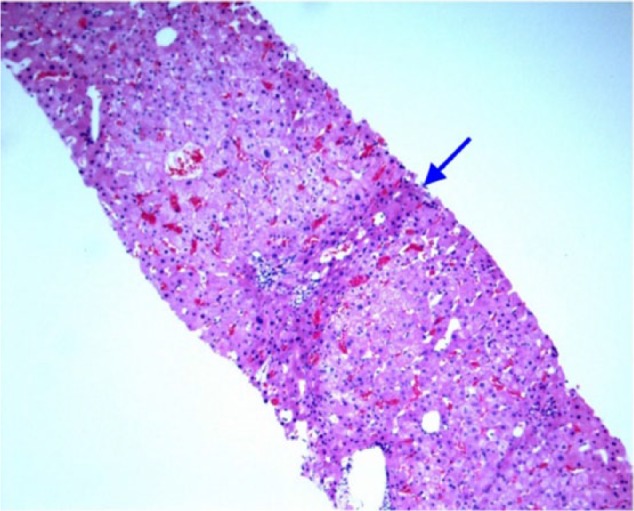
Hematoxylin and eosin stain of liver biopsy. The hepatocytes between the nodules are compressed and atrophic (blue arrow). The liver biopsy slide does not belong to the case reported in this article as its slides were accidentally damaged under unexpected circumstances. The slide is provided to clearly illustrate the histologic features of nodular regenerative hyperplasia.

**Figure 2.**
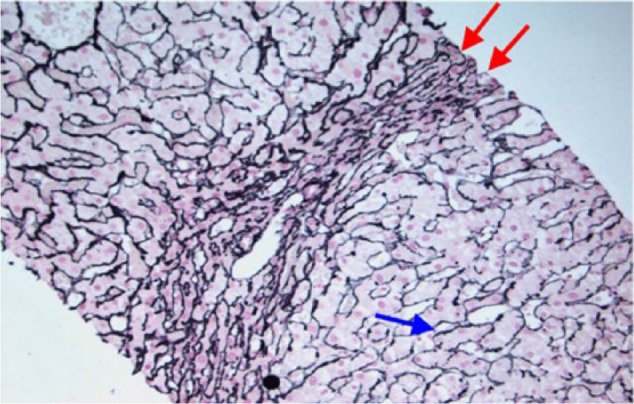
Reticulin stain of liver biopsy. The reticulin network is compressed in the parenchyma between the nodules (red arrows). Hepatocytes within nodules are arranged in plates that are 1 to 2 cells thick (blue arrow). The liver biopsy slide does not belong to the case reported in this article as its slides were accidentally damaged under unexpected circumstances. The slide is provided to clearly illustrate the histologic features of nodular regenerative hyperplasia.

Initial computed tomography scan of abdomen and pelvis at that time showed splenomegaly measuring 18.3 cm × 10.9 cm on coronal series and large collateral vessels suggestive of portal hypertension without associated cirrhosis. Esophagogastroduodenoscopy was significant for large esophageal varices without high-risk stigmata. Liver biopsy showed minimal fibrosis around the portal areas with no significant inflammation. The lobules showed focal zones of thin hepatocyte plates on reticulin stain with adjacent areas showing mild regenerative changes (Figures 1 and 2). The diagnosis of NRH was made and patient was placed on propranolol 10 mg twice a day. Two years after the diagnosis of NRH, the patient presented with hematemesis without hemodynamic instability and was found to have active gastric variceal bleeding. Consequently, she underwent transjugular intrahepatic portosystemic shunt (TIPS). Portal pressure gradient decreased from 36 mm Hg to 13 mm Hg. Postprocedure course was complicated by mild encephalopathy controlled with lactulose.

**Figure 3.**
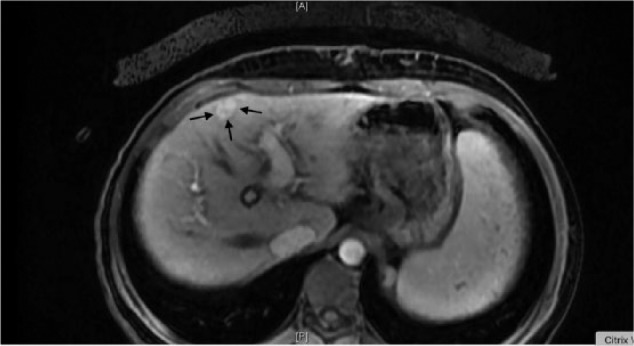
Hepatocellular carcinoma (arrows) with arterial enhancement (arterial phase).

**Figure 4.**
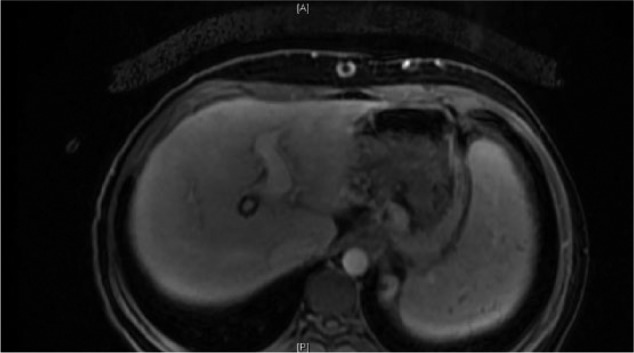
Washout during the venous phase.

**Figure 5.**
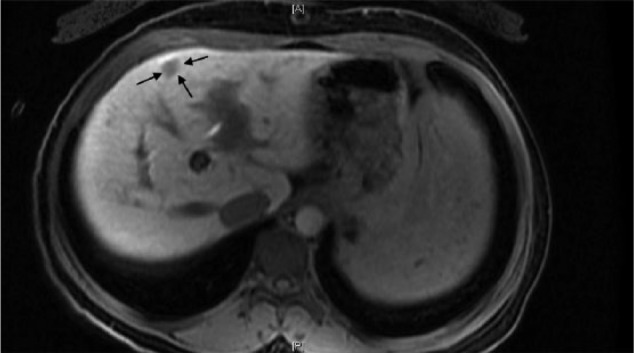
Hepatocellular carcinoma (arrows) on hepatobiliary phase.

Magnetic resonance imaging performed for pre-TIPS evaluation of the portal vasculature showed a 7-mm hypervascular nodular focus in segment IVa. Follow-up magnetic resonance imaging performed 4 months post-TIPS placement showed stable lesion suspicious for early HCC; however, 6 months later it showed an increase in nodule size to 1.7 cm × 1.3 cm, consistent with HCC (Figures 3-5, courtesy of Dr. Victoria Cherniak, Bronx, NY). Liver Imaging Reporting and Data System (LI-RADS) score was 4, which refers to hyperenhancement on the imaging arterial phase, a finding that is consistent with probable HCC. Alpha-fetoprotein level was 1.9 IU/mL. The case was comprehensively discussed in our transplant selection multidisciplinary meeting and was deemed to be a candidate for liver transplantation. The patient expressed interest in liver transplantation and was placed on the transplant waiting list with MELD score of 11. Since the lesion was LI-RADS 4, the recommended approach is that there is no need for biopsy and to monitor the lesion radiologically. Three months later, the patient decided to have the intrauterine device removed due to bleeding, and she opted not to have other forms of contraception provided. She is currently pregnant and has been following with maternal-fetal medicine specialist. Relisting for liver transplantation is pending antepartum and postpartum clinical course. In the meantime, her HCC has been monitored with serial imaging without further intervention given its size of less than 2 cm.

## Discussion

Increasing amount of literature has established the relationship between NRH and antiretroviral, antineoplastic, and immunosuppressive medications.^3,5^ Well-described agents include azathioprine, mercaptopurine, thioguanine, didanosine, stavudine, and oxaliplatin. One study showed that cumulative exposure to didanosine, stavudine, tenofovir, combinations of didanosine with stavudine, and didanosine with tenofovir was longer in HIV seropositive patients with NRH compared to HIV seropositive patients without NRH.^3^ Another study reported 8 HIV seropositive patients who were referred for evaluation of abnormal liver enzymes, and all were found to have NRH and all were exposed to didanosine, and some to stavudine and zidovudine for 1 to 2 years.^5^ Following cessation of offending medications, several case reports have documented improvement of liver enzymes and platelet counts 6 weeks to 2 years after cessation.^5^

The clinical presentation of NRH in HIV seropositive patients is similar to the non-HIV population. One meta-analysis of 95 cases of NRH in HIV patients showed that the diagnosis was most frequently prompted in asymptomatic patients with incidental lab abnormalities.^1^ Mild abnormalities in liver tests were most commonly noted, while the synthetic function was well preserved. The most common manifestations of portal hypertension were esophageal varices (66 of 95), followed by ascites (30 of 95) and splenomegaly^1^ (25 of 95). While esophageal varices were identified in 66 of 95 cases, only 28 patients developed gastrointestinal bleeding in the setting of esophageal varices. All patients with evidence of esophageal varices were placed on nonselective beta-blockers. Similar findings have been seen in patients with NRH without HIV infection, except that patients with coexisting NRH and HIV have been reported to have higher incidence of ascites compared to NRH alone.^1^

The data about incidence of HCC in patients with NRH are very limited. NRH is not considered a premalignant condition; however, some literature suggested a temporal relationship between HCC and NRH.^12-14^ Nzeako et al reviewed 342 noncirrhotic patients, of which 23 patients had NRH, and the authors found that a significantly greater portion of those with NRH had liver cell dysplasia compared with those without NRH.^13^ Kobayashi et al surveyed NRH in 11 autopsied noncirrhotic livers with HCC and identified one case of NRH with history of antineoplastic infusion premortem.^14^ Sood et al reported a case of de novo HCC in patient with NRH found on screening for HCC.^15^ Our case represents the first case report on de novo HCC in an HIV-positive patient with NRH.

The treatment of NRH has been focused on supportive and preventive measures with nonselective beta-blockers and/or variceal ligation in patients with evidence of large varices. For patients who have refractory variceal bleeding, ascites or encephalopathy despite variceal ligation, TIPS, and medical management, respectively, liver trasnplantation may be indicated. One meta-analysis identified 17 studies in which liver transplantation was considered as a treatment for NRH with severe portal hypertension refractory to conventional therapies.^16^ Twenty-six patients received liver transplantation. The most frequent indications were esophageal variceal bleeding (17/26; 65.3%), ascites (16/26; 61.5%), encephalopathy (8/26; 30.7%), and spontaneous bacterial peritonitis (1/26; 3.8%). The 5-year patient and graft survival rate was 78.3%.^16^ Although liver transplantation is a rarely needed treatment modality in patients with NRH, the clinical presentation of our patient with HCC in the setting of decompensated liver disease makes liver transplantation is the treatment of choice.

In conclusion, this case report describes a unique clinical scenario of de novo HCC in an HIV-positive patient with NRH. Until the association is further observed and proven, it may be reasonable to periodically screen patients with NRH for HCC.
